# Is ageism among prescribers an influential factor in the management of depression in the elderly? A vignette-based study

**DOI:** 10.1093/fampra/cmag003

**Published:** 2026-03-03

**Authors:** Amina Stolz, Julie Pluies, Setareh Ranjbar, Beatriz Pozuelo Moyano, Stéphane Mouchabac, Pierre Vandel, Stéphane Adam, Jean-Pierre Schuster

**Affiliations:** Department of Psychiatry, Service of Old Age Psychiatry, Lausanne University Hospital and University of Lausanne, Prilly 1008, Switzerland; Department of Psychiatry, Service of Old Age Psychiatry, Lausanne University Hospital and University of Lausanne, Prilly 1008, Switzerland; Department of Psychiatry, Psychiatric Epidemiology and Psychopathology Research Center, Lausanne University Hospital and University of Lausanne, 1008 Prilly, Switzerland; Department of Psychiatry, Service of Old Age Psychiatry, Lausanne University Hospital and University of Lausanne, Prilly 1008, Switzerland; ICRIN Psychiatry (Infrastructure of Clinical Research in Neurosciences-Psychiatry), Sorbonne Université, Brain Institute (Institut du Cerveau et de la Moëlle (ICM)), Institut National de la Santé et de la Recherche Médicale (INSERM), Centre National de la Recherche Scientifique (CNRS), 75013 Paris, France; Department of Psychiatry, Saint-Antoine Hospital, Sorbonne University, 75012 Paris, France; Department of Psychiatry, Service of Old Age Psychiatry, Lausanne University Hospital and University of Lausanne, Prilly 1008, Switzerland; Department of Psychology, Psychology of Aging Unit, University of Liège, 4000 Liège, Belgium; Department of Psychiatry, Service of Old Age Psychiatry, Lausanne University Hospital and University of Lausanne, Prilly 1008, Switzerland

**Keywords:** general practice, depressive disorder, ageism, decision-making, psychotherapy, antidepressive agents

## Abstract

**Background:**

The decision-making process, from diagnosing depression to treatment proposal, involves many interrelated factors. Patient age has been identified as a factor that influences care proposals. Our aim is to investigate the association between how prescribers perceive ageing and their decision-making when treating depression.

**Methods:**

A cross-sectional survey of 57 physicians was conducted. The questionnaire collected socio-demographic data, and ageing semantic differential (ASD) scale to assess ageism. It also examined the reactions to treatment proposals in a clinical vignette about an 82-year-old woman with a single-episode depressive disorder of unspecified severity (mild or moderate). Physicians' reactions to two treatment options—antidepressant medication and psychotherapy—were assessed independently. For each option, participants could indicate whether they were in favor of initiation, against initiation, or had no opinion. Multiple linear regression was used to study the association between ASD and attitudes toward the initiation of antidepressant medication and psychotherapy.

**Results:**

Prescribers' representations of ageing were significantly associated with their therapeutic decisions for late-life depression. When presented with the same clinical vignette, physicians showed substantial variability in treatment preferences. After adjustment, physicians in favor of initiating psychotherapy exhibited significantly more negative views of ageing compared with those opposed to this option. No significant association was observed between ageist attitudes and the decision to initiate antidepressant treatment.

**Conclusions:**

The research underscores the complex role of ageism in medical decision-making and highlights the need for targeted training to mitigate its impact on late-life depression care.

## Introduction

Primary care systems are currently facing major challenges, notably the ageing of the population and the increasing prevalence of mental health disorders. In many healthcare systems, general practice is the cornerstone of care service provision for depression in the elderly [1, 2].

The decision-making process, from diagnosis to treatment proposal, involves many interrelated factors. Some of these factors are related to the patients, such as their symptoms and comorbidities, demographic characteristics, expectations, perceived seriousness of the disease, and perceived usefulness of treatments. Other factors are related to the practitioners, such as professional experience. There are also shared factors, such as the doctor–patient relationship, the organization of the healthcare system, and the availability and cost of therapies [3-7].

Initiating treatment for depression management in primary care consultations is a complex medical act. Prescribing such treatment is typically the outcome of a shared decision-making process between the general practitioner (GP) and the patient [8]. Considering specifically the patient's characteristics and their impact on the therapeutic management of depression, the age of the patient stands out. Physicians reported feeling less prepared to identify and treat older patients than younger ones [9]. Physicians consider that elderly patients are less likely to benefit from pharmacotherapy, psychotherapy, or referral to a specialist than younger patients with depression or suicidal ideation [9-11].

In European societies, age is a major factor of discrimination, known as ageism [12]. Butler originally proposed this term to describe all forms of discrimination, segregation or contempt based on age [13]. Age-related stereotypes can negatively impact caregivers' perceptions of the elderly, leading to biased therapeutic decisions [14, 15]. Although age has been identified as a factor influencing care proposals for depression [9-11], to our knowledge, no study has examined the direct link between the prescriber's representation of ageing and the therapeutic decision-making for a single depressive episode.

We aim to investigate the association of the prescriber's representations of ageing and the therapeutic decision-making for a depressive episode, i.e. on physicians' propensity to prescribe antidepressants and psychotherapy for single depressive episodes.

## Materials and methods

Our study is based on data from a cross-sectional survey of physicians (GPs and geriatricians). Physicians were invited via email to complete an anonymous self-administered questionnaire. The electronic questionnaire was accessible via a link provided in the email. Email contact lists were generated by the authors based on publicly accessible professional directories and institutional listings of healthcare professionals. Participation was voluntary, and no random sampling strategy was used.

The questionnaire was designed by the authors of the study. It consists of three parts. The first parts requested socio-demographic data, including: sex, age, doctor's profession (GP or geriatrician), and the country in which doctors practice (France or Switzerland). The second part consists of the ageing semantic differential (ASD) scale. The third part is based on questions related to a vignette.

To measure participants' attitudes and stereotypes toward older people, we used the French version of the ASD scale [16]. The ASD is a self-assessment instrument consisting of 26 items composed of matched pairs of adjectives with opposite meanings (e.g. active–passive, flexible–rigid, and independent–dependent). Participants were asked to indicate how they judged elderly persons on a seven-point Likert scale for each matched pairs of adjectives proposed. The total score corresponds to the sum of all the items. A high score corresponds indicates a negative view of the elderly (ageism). A score of 78 corresponds to neutrality score (min = 26 − max = 182).

The final part of the questionnaire consisted of a clinical case and related questions. The traditional vignette method consists of two main elements: (i) the creating and implementing vignettes; and (ii) the gathering of detailed information about participants' specific characteristics, when analyzing the vignette data. Typically (and in our research), vignettes are typically concise, hypothetical scenarios designed to elicit reactions. Vignettes allow the extraction and examination of participants' beliefs, attitudes, and judgments in a manner distinct from conventional questionnaires, as they are set within a context rather than treated in isolation. We adhere to the recommendations for vignettes applicable for research purposes. For example, we avoid placing the participants in the vignettes [17, 18].

The vignette describes an 82-year-old woman visiting her GP. She consults at the request of her daughter, who accompanies her. The patient was widowed 4 years ago and lives independently. She has three children and one of whom, her daughter lives nearby and occasionally helps her with groceries. She suffers from bilateral hearing loss and treated hypertension. She does not consume alcohol or tobacco. She has no notable medical history. According to the clinical history, she has experienced a change in her overall level of activity for about a month. The patient reports fatigue, early morning awakening, sadness, feeling down, hopelessness about the future, and reduced in daily activities. However, she still enjoys certain activities, such as listening to music and spending time with her grandchildren. There are no psychotic elements and no suicidal ideations. Her weight is stable. The patient has no cognitive complaints, and her Mini Mental State examination score is 27/30. The clinical examination, blood work, and electrocardiogram are unremarkable. She has been on the same treatment for a year, consisting of a taking daily intake of an angiotensin-converting enzyme inhibitor. According to the ICD 11, this is a single-episode depressive disorder of unspecified severity 6A70.5. (mild or moderate) [19]. The doctor recommends initiating antidepressant and psychotherapeutic treatments.

Participants were asked to evaluate each treatment option independently. For both antidepressant medication and psychotherapy, three response options were available: in favor of initiation, against initiation, or no opinion. No explicit “watchful waiting” or “no specific treatment” option was proposed.

A pilot study with 7 participants was conducted to review the content. Based on the feedback, revisions were made to the initial questionnaire, to render the clinical vignette as realistic as possible and to weigh up the amount of information necessary for proposing a therapeutic approach.

Descriptive analyses were used to describe the sample characteristics: mean (SD) for continuous variables and count (percentage) for categorical variables. We subsequently divided the population according to their therapeutic attitude (prescription of an antidepressant or not and prescription of psychotherapy or not). The sample characteristics were then compared between these groups using Kruskal–Wallis rank sum test and Fisher's exact test for continuous and categorical variables, respectively.

Multiple linear regression models were performed to examine the association between the ASD scale (as dependent variable), and reactions to treatment proposals (antidepressant and psychotherapy) in a clinical vignette of an 82-year-old woman with a single-episode depressive disorder of unspecified severity in three categories (exposure of interest). The models controlled for sex, age, doctor's profession (GP or geriatrician), and country of practice (France or Switzerland). The results of the regressions are reported as beta coefficients and 95% confidence intervals. We used a *P*-value <.05 was used as the significance threshold for all analyses. We analyzed the data using R.4.4.1.

An ethics approval was not required for the current study as per applicable institutional and national guidelines. Data were collected via an anonymous online questionnaire. We have no information about the participants other than their sex, age, profession and country of practice. Participants were informed that the results of the survey would be used for research purposes. By completing the questionnaire, participants consented to the use of their anonymous responses for research purposes. No incentives were offered.

## Results

Of the 57 physicians involved in the study, 54% were women. The mean age of the respondents was 46-years-old (SD = 10). Participants exercised in two countries: in Switzerland (75%) and in France (25%). The survey population consisted of family physicians (61%) and geriatricians (39%). The total mean score of the ASD-26 in this population was 100 (SD = 15).

When asked about the vignette and the GP's therapeutic choices, the practitioners had various opinions (Table 1). 28% agreed of initiating an antidepressant and 55% agreed of initiating psychotherapy. 17% did not have an opinion on the therapeutic choice of initiating antidepressant treatment, and 15% did not have an opinion on initiating psychotherapy (Table 1).

**Table 1:** Classification of practitioners based on their attitude toward therapeutic proposals (N = 57).

		Attitude toward the initiation of antidepressant			
		+^a^	0^b^	−^c^	*P*-value^d^
Attitude toward the initiation of psychotherapy	+^a^	10 (18%)	5 (9%)	16 (28%)	.09
	0^b^	4 (7%)	3 (5%)	2 (3%)	
	−^c^	2 (3%)	2 (3%)	13 (23%)	

Attitude toward the initiation of a treatment (antidepressant or psychotherapy).

^a^In favor of initiation.

^b^No opinion on whether or not to initiation.

^c^Unfavorable to the initiation.

^d^Fisher's exact test.

There were no differences in terms of sex, age, profession, or country of practice between the groups in favor of or opposed to initiating an antidepressant (Tables 2 and 3). The group in favor of initiating psychotherapy, was significantly younger than the group not in favor of this treatment and had a more negative viewing of ageing (ASD score). Doctors practicing in France, compared with those practicing in Switzerland, were significantly more likely to be in favor of initiating psychotherapy (Table 4). After adjusting for sex, age, profession, and country of practice, the group in favor of initiating psychotherapy compared with the group against this option had a more negative view of ageing than the group against this option (β = 10.58, IC = 1.01–20.16, *P* = .031) (Table 5).

**Table 2:** Characteristics of practitioners with respect to therapeutic attitude toward the initiation of antidepressant.

		Overall population	AD+	AD 0	AD−	*P*
*N*		57	16	10	31	
**Sex; *n* (%)**						.5^a^
	F	31 (54%)	7 (44%)	5 (50%)	19 (61%)	
	M	26 (46%)	9 (56%)	5 (50%)	12 (39%)	
**Age (years)**	Mean (SD)	46 (10)	46 (11)	49 (12)	45 (9)	.6^b^
**Profession; *n* (%)**						.4^a^
	GP	35 (61%)	10 (63%)	8 (80%)	17 (55%)	
	Geriatrist	22 (39%)	6 (38%)	2 (20%)	15 (45%)	
**Country of practice; *n* (%)**						.6^a^
	Switzerland	43 (75%)	12 (75%)	9 (90%)	22 (71%)	
	France	14 (25%)	4 (25%)	1 (10%)	9 (29%)	
**ASD**	Mean (SD)	100 (15)	103 (17)	99 (18)	99 (13)	.8^b^

AD+: in favor of initiation an antidepressant; AD−: unfavorable to the initiation of an antidepressant; AD 0 : no opinion on whether or not to initiation an antidepressant.

^a^Fisher's exact test.

^b^Kruskal–Wallis rank sum test.

**Table 3:** Multiple linear regression to study the association between ASD and attitude toward the initiation of antidepressant (*N* = 57).

Predictors	β	CI	*P*-value
**Sex (male)**	6.92	−1.62–15.47	.110
**Age**	−0.08	−0.50–0.34	.713
**Profession (GP)**	2.27	−10.14–14.68	.714
**Country of practice (Switzerland)**	−5.49	−19.29–8.32	.428
**AD− (ref.)**			
**AD+**	5.11	−4.35–14.58	.283
**AD 0**	1.55	−9.82–12.91	.786
***R* ^2^**	0.087		

AD+: in favor of initiation an antidepressant; AD−: unfavorable to the initiation of an antidepressant; AD 0 : no opinion on whether or not to initiation an antidepressant (ref: AD−). The model is adjusted for sex, age, profession, and country of practice.

**Table 4:** Characteristics of practitioners with respect to therapeutic attitude toward the initiation of psychotherapy.

		Overall population	Psycho+	Psycho 0	Psycho−	*P*
*N*		57	31	9	17	
**Sex; *n* (%)**						>.9^a^
	F	26 (46%)	15 (48%)	4 (44%)	7 (41%)	
	M	31 (54%)	16 (52%)	5 (56%)	10 (59%)	
**Age (years)**	Mean (SD)	46 (10)	43 (10)	50 (12)	50 (8)	.**035**^b^
**Profession; *n* (%)**						.12^a^
	GP	35 (61%)	16 (52%)	8 (89%)	11 (65%)	
	Geriatrist	22 (39%)	15 (48%)	1 (11%)	6 (35%)	
**Country of practice; *n* (%)**						**.021** ^a^
	Switzerland	43 (75%)	19 (61%)	9 (100%)	15 (88%)	
	France	14 (25%)	12 (39%)	0 (0%)	2 (12%)	
**ASD**	Mean (SD)	100 (15)	103 (14)	106 (11)	92 (16)	**.029** ^b^

Psycho+: in favor of initiation a psychotherapy; Psycho−: unfavorable to the initiation of a psychotherapy; Psycho 0: no opinion on whether or not to initiation a psychotherapy. Bold values indicate statistically significant results (*P* < .05).

^a^Fisher's exact test.

^b^Kruskal–Wallis rank sum test.

**Table 5:** Multiple linear regression to study the association between ASD and attitude toward the initiation of psychotherapy (*N* = 57).

Predictors	β	CI	*P*-value
**Sex (male)**	7.01	−1.09–15.10	.088
**Age**	0.01	−0.41–0.43	.973
**Profession (GP)**	0.73	−11.05–12.50	.902
**Country of practice (Switzerland)**	−2.68	−16.12–10.76	.690
**Psy− (ref.)**			
**Psy+**	10.58	1.01–20.16	.**031**
**Psy 0**	13.68	1.67–25.68	.**026**
***R* ^2^**	0.183		

Psy+: in favor of initiation a psychotherapy; Psy−: unfavorable to the initiation of a psychotherapy; Psy 0 : no opinion on whether or not to initiation a psychotherapy (ref: Psy−). The model is adjusted for sex, age, profession, and country of practice. Bold values indicate statistically significant results (*P* < .05).

## Discussion

This study contributes to the growing body of literature on ageism and depression in later life by highlighting the role of prescribers' perceptions of ageing in shaping therapeutic decision-making. Whereas prior research has largely focused on age-related disparities in access to or rates of treatment, our findings extend this work by directly examining the association between physicians' ageist attitudes, measured using a validated ageism scale, and their treatment preferences, assessed through a vignette-based methodology. Importantly, the observed association between higher levels of ageism and an increased likelihood of recommending psychotherapy nuances prevailing assumptions that ageism necessarily translates into therapeutic nihilism, and suggests a more complex influence of age-related beliefs on clinical practice.

Our study highlights the great variability in physicians' attitudes toward treatment despite the assessment of the same clinical situation, i.e. a patient consulting with a single episode of depressive disorder of, unspecified severity 6A70.5. (mild or moderate). Just over one-quarter of participants opted to initiate antidepressant treatment, while slightly more than half recommended psychotherapy. For comparison, a study conducted with Swiss GPs found that, when presented with the case of a 42-years-old woman female with mild depression, 76% recommended watchful waiting, 11% recommended antidepressant treatment, and 40% recommended psychotherapy [5 ].

Our main finding is the link between ageism and therapeutic decision-making. We did not find evidence supporting a link between prescribers' ageist attitudes and the likelihood of prescribing antidepressants. Therefore, our results do not support the hypothesis that negative perceptions of ageing influence the decision to initiate pharmacotherapy. However, we did observe an unexpected association: the more negative a doctor's view of ageing is, the more likely they are to prescribe psychotherapy, for a mild-to-moderate single depressive episode. The unexpected link between negative views of ageing (ageism) and the prescription of psychotherapy for mild-to-moderate depression raises questions.

Ageism is a complex, multifaceted phenomenon. Rooted in often unconscious prejudices, ageism expresses itself in everyday interactions and impacts our care practices. For instance, the common belief that elderly depression in the elderly is a natural consequence of ageing can lead to an underestimation of the diagnose of depression, or even to therapeutic nihilism [20]. While psychotherapy is a recommended and appropriate treatment in the present case, its prescription in this context may be shaped by ageist assumptions. Our study focuses on the therapeutic decisions, and we have no information about the GP's thoughts behind these decisions. Thus, ageism may not directly affect the final treatment choice, but rather influence earlier stages of the care process, such as the diagnostic interpretation or expectations regarding the patient's capacity for recovery. Ageist beliefs may lead physicians to underestimate older adults' ability to recover or to perceive their emotional suffering as inevitable. Moreover, such beliefs could impact how effective psychotherapy is perceived to be in this population. Some studies suggest that clinicians are less confident in the efficacy of psychotherapy for older patients [21], even though evidence shows that older adults can indeed benefit from such interventions [22, 23]. The association we observed—between higher levels of ageism and a greater likelihood of prescribing psychotherapy—may reflect a perception of psychotherapy as a form of emotional support rather than a robust, evidence-based treatment for depression. In this light, psychotherapy could be viewed by some practitioners less as a clinical intervention and more as a palliative measure.

Beyond the hypothesis that psychotherapy may be perceived as a form of emotional support, several complementary mechanisms may help explain this association. Physicians holding more negative views of ageing may perceive older adults as medically fragile, at greater risk of adverse drug reactions, and more difficult to treat effectively [9, 10, 20]. In this context, psychotherapy may be regarded as a “safer” or less burdensome option. Such reasoning reflects the idea that ageism may manifest not only as therapeutic nihilism but also through paternalistic attitudes, whereby clinicians favor what they perceive as the least risky intervention [20]. These tendencies may be reinforced by lower prescriber confidence in diagnosing and treating late-life depression [9, 21, 22]. Together, uncertainty and ageist assumptions may therefore steer clinical decisions toward psychotherapy, not necessarily because it is viewed as optimal, but because it is perceived as carrying fewer risks.

Several limitations in our study should be acknowledged. First, the relatively small sample size limits the generalizability of our findings. Physicians were recruited via email using contact lists generated from publicly available professional directories. As no random sampling strategy was applied, the sample may not be fully representative of the broader physician population, and selection bias cannot be ruled out. Additionally, some key data were missing. For example, we do not know whether participants work in urban or rural areas. In France, however, GP practices located in urban areas were found to be associated with higher levels of antidepressant prescriptions [24].

Second, to characterize ageism, we used the ASD, which measures explicit ageism, but does not consider its implicit dimension [25]. Nevertheless, it is noteworthy that the mean ageism score in our sample was above the neutral threshold. This finding is consistent with previous studies, which have repeatedly reported more negative representations of ageing among healthcare professionals [14].

Third, we used an online vignette-based methodology to evaluate doctors' attitudes [17]. Although vignette methodology has strengths, it also has limitations. Most notably, our study was not a real-life evaluation, and the clinical vignette used in our study was incomplete. This makes it impossible to characterize with any certainty the degree of severity of the depressive episode. Clinical guidelines distinguish between management strategies for mild and moderate depression, particularly with regard to the use of antidepressants and psychotherapy [1]. As the vignette did not clearly differentiate between severity levels, it may have introduced legitimate ambiguity into the clinical reasoning process. Consequently, some variability in treatment proposals reflects appropriate clinical judgement rather than ageist attitudes alone. In this context, selecting “no opinion” may represent a cautious, evidence-based approach rather than indecision. Although we could not model “no opinion” as a distinct outcome, future studies should examine this group more explicitly.

Furthermore, this methodology enabled us to identify attitudes, but not necessarily behaviors. To minimize this bias, participants were given the option to respond neutrally, indicating neither support nor opposition to the introduction of a therapeutic measure. Furthermore, the vignette methodology disregards the alliance between physicians and their patients, even though this is acknowledged as an important condition for family doctors to recognize and manage depression [7, 26].

Finally, the relatively low *R*²-values observed in our models suggest that ageism and the included covariates account for only a limited proportion of the variance in therapeutic decisions, consistent with the multifactorial nature of clinical decision-making in late-life depression. Several potentially influential factors were not assessed, including patient characteristics (such as treatment preferences, social support, and socioeconomic vulnerability), and contextual constraints like access to psychotherapy. Physician-related factors, including clinical experience and confidence in managing late-life depression, may also contribute meaningfully [1, 27]. Collectively, these unmeasured variables may explain why ageism alone accounts for only a small share of the observed variability in therapeutic attitudes.

The relationship between ageism and the management of depression in older adults is complex and multifaceted. A prescriber's perception of ageing may influence their clinical decisions, including whether to recommend psychotherapy as a treatment option. Ageism has no place in healthcare, and efforts must be made to ensure that it does not influence care practices. Further research is needed to achieve this goal, and to explore how physicians perceive ageing, and how these perceptions influence their treatment choices and expectations regarding of therapeutic outcomes. With this knowledge, healthcare professionals will be better equipped to recognize and challenge their own biases, leading to person-centered care. Comprehensive education and training programs that address ageism and the specificities of late-life depression are essential.

## Data Availability

The datasets used and analyzed during the current study are available from the corresponding author upon reasonable request.

## Artificial intelligence

During the preparation of this work, the authors used ChatGPT by OpenAI to improve language and readability. After using this tool, the authors reviewed and edited the content as needed and take full responsibility for the content of the published article.
